# Fracture propagation and water seepage behavior in overburden strata at Tunbao coal mine

**DOI:** 10.1038/s41598-025-29348-w

**Published:** 2025-11-29

**Authors:** Jiaxu Dong, Hao Liu, Wenbin Sun, Xinxue Liu, Zhijie Tao

**Affiliations:** 1 College of Energy and Mining Engineering, Shandong University of Science and Technology, Qingdao, 266590 PR China; 2 Mine Disaster Prevention and Control-Ministry of State Key Laboratory Breeding Base, Shandong University of Science and Technology, Qingdao, 266590 PR China; 3 College of Safety and Environmental Engineering, Shandong University of Science and Technology, Qingdao, 266590 PR China

**Keywords:** Overburden strata fracture, Water seepage behavior, Hydraulic properties, Failure prediction, Energy science and technology, Engineering, Natural hazards

## Abstract

This study investigates water inrush mechanisms in goaf roof strata through multi-scale analysis of fracture network evolution and seepage dynamics. A three-zone overburden model was established via PFC3D simulation and validated with experimental data. Coupled PFC-CFD simulations reveal that seepage is governed by the interplay of pressure gradients, fracture pathways, and flow rates. Key findings include: nonlinear attenuation of pressure gradients amplifies flow velocities in dominant channels; increased injection rates widen effective channels by 86%; and high-velocity flow enhances permeability through erosion, resulting in significant directional anisotropy. These results provide insight into the positive feedback mechanisms controlling dynamic seepage in fractured rock masses.

## Introduction

As a key energy pillar in the world, the mining intensity of coal continues to increase with the growth of energy demand, but high-intensity mining is easy to cause overburden damage and form a large number of water-conducting fracture zones. This kind of fractured zone may be connected with the aquifer, inducing the roof water inrush accident in the goaf, causing serious casualties, equipment damage and ecological environment damage, which has become the core water hazard that restricts the safe and efficient mining of coal mines. Therefore, it is the key to accurately prevent and control the risk of roof water inrush and ensure the safety of coal mine production to clarify the evolution characteristics of overlying rock fissures in goaf, reveal the law of water seepage and identify the dominant seepage channels, which is of great practical significance to promote the development of mine disaster prevention theory and engineering practice.Therefore, simulating fracture evolution and seepage patterns, identifying dominant flow pathways, and accurately predicting water leakage are essential for managing and mitigating water inrush risks in goaf roofs.

Recent studies have advanced the understanding of fracture zone development in overlying strata, providing theoretical foundations for mining safety and disaster prevention. Academician Song^1^ established a rock movement-based theory integrating prediction, control, and evaluation of ground pressure. Yu and Cheng^2^ et al. applied resistivity methods to estimate fracture zone height, while Cui^3^ combined borehole data, theoretical calculations, and numerical simulations to quantify fracture development. Wang Qi^4^ used theoretical analysis and numerical simulation to study the development law of overburden rock cracks in composite goaf during excavation and mining. Li Jianwen et^5^al.proposed a fuzzy comprehensive evaluation model of rock mass integrity based on combination weighting for the lack of fracture development in the evaluation of rock mass integrity in coal mine goaf. By designing the seepage test of coal ( rock ) samples, Zhi et^6^ al.obtained the change function of permeability of coal ( rock ) samples with vertical stress, and revealed the distribution law of permeability of porous media in goaf. Gao Mingzhong et^7^ al.explored the mining dynamic behavior and seepage evolution law of coal rock. Under the action of water, the micro surface of coal rock appears spalling phenomenon, and the micro cracks are expanded and developed. Gao et al.^8^studied the macroscopic and microscopic mechanical behaviors and seepage characteristics of coal under hydro-mechanical coupling. Hao et al.^9^ conducted extensive experimental studies on the water-gas seepage characteristics of compacted crushed coal rocks including siltstone, medium grained sandstone, fine grained sandstone and coal and obtained the water seepage laws in the coal rock samples with single particle sizes and graded particle sizes in goaf. The compactions of different combined crushed coal rocks were carried out respectively, and the relationship between permeability and axial pressure was revealed by the seepage experiments on the compacted coal rocks of different combinations.

Although the existing research has laid a foundation for the study of overburden fracture evolution and seepage characteristics from multiple dimensions such as theory, experiment and numerical simulation, most of the research relies on traditional numerical methods such as FLAC3 D or physical simulation. It is difficult to accurately describe the dynamic initiation, expansion, penetration and network formation process of fractures in the mining process from the particle scale. There is a deviation between the description of the spatial distribution characteristics, development height and connectivity of fractures and the actual working conditions in the field, and it is difficult to support the accurate identification of dominant seepage channels. Taking the goaf of WⅡ02040502 fully mechanized caving face in Tun bao Coal Mine as an example, we studied the collapse of overburden, fracture distribution and seepage law in goaf by PFC numerical simulation with the focus on the fracture evolution and water seepage law during coal seam caving and the water inrush caused by the development of fracture networks in overburden, and verified the results with the similar simulation experiments and field observations. Our study aimed to more accurately understand the collapse law, porosity and water seepage characteristics of overburden, provide a basis for preventing and controlling water inrush from the roof of goaf and establishing a prevention and early warning system, and ensure safe and efficient mining in Tun bao Coal Mine.

## Evolution characteristics of overburden collapse and porosity in Goaf

### Overview of geological characteristics of Tun Bao coal mine

**Fig. 1:**
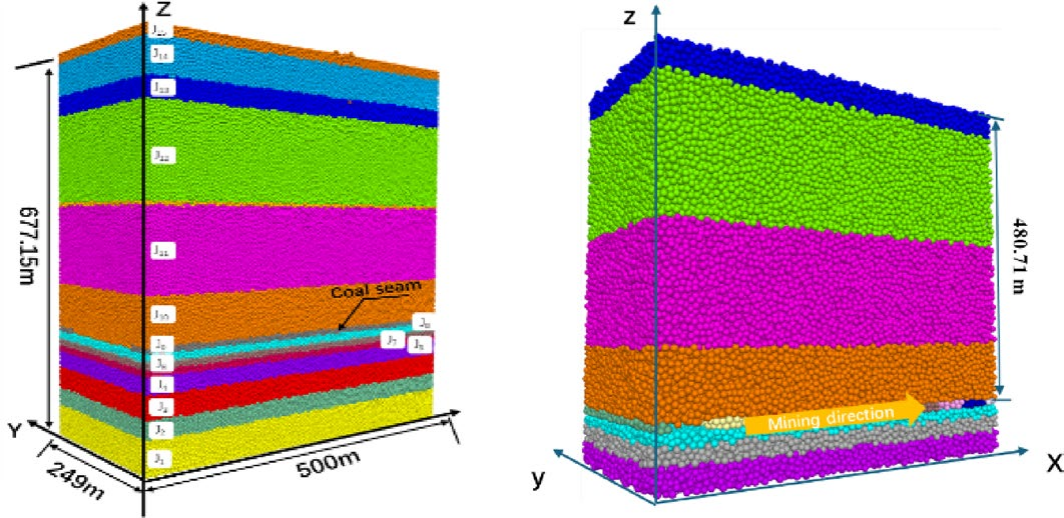
Schematic diagram of the goaf model.

The object mine, Tun bao Coal Mine, is located in Xinjiang Mining Area, China. Figure 1 shows the distribution of overburden in the WII02040502 working face of Tun bao Coal Mine, mapped according to the relevant geological data. the outburst proneness of the M4-5 coal seam and its roof strata is weak. In the mine with rock burst coal seams, the roof coal seam is usually loose and broken, and is prone to collapse upon the appearance of mine pressure. The advance of the working face causes complex developments of multi-layer fracture channels and uncertainty in the seepage law, and the diversion fracture zone tends to merge with the water bearing strata, which likely lead to water inrush from the goaf roof.

### PFC numerical simulation

#### Selection of macroscopic and microscopic parameters

There are two particle bond models, e.g., contact bond model and parallel bond model, available in the PFC software. In the parallel bond model, stiffness is contributed by both contact stiffness and bond stiffness. It can simulate coal more realistically during tensile or shear fracture^10^. Therefore, the parallel bond model is adopted in this work for the simulation. The relationship between the macroscopic and microscopic parameters is determined based on the particle flow theory. The macroscopic mechanical parameters of coal are converted into the microscopic parameters for the simulation using the empirical formulas below.

Elastic modulus empirical formula,

1 $$E=\frac{\delta }{\varepsilon }$$

2 $$E/{E_c}=a+b\ln ({k_n}/{k_s})$$

where *E* is the elastic modulus, GPa; σ is normal stress, GPa;ε is a positive strain; *E*_*c*_ is Young’s modulus, GPa; *k*_*n*_*/k*_*s*_ is the stiffness ratio; a = 1.652; and b=-0.395.

Poisson’s ratio empirical formula,

3 $$\:v=c{ln}({k}_{n}/{k}_{s})+d$$

where *v* is Poisson’s ratio; c = 0.209; and d = 0.111.

Regression of uniaxial compressive strength.

4 $$\:\frac{{\sigma\:}_{c}}{\overline{\sigma\:}}=\left\{\begin{array}{c}a{\left(\frac{\overline{\tau\:}}{\overline{\sigma\:}}\right)}^{2}+b\frac{\overline{\tau\:}}{\overline{\sigma\:}},\hspace{0.33em}\hspace{0.33em}\hspace{0.33em}\hspace{0.33em}0<\frac{\overline{\tau\:}}{\overline{\sigma\:}}\le\:1\\\:c\hspace{0.33em}\hspace{0.33em}\hspace{0.33em}\hspace{0.33em}\hspace{0.33em}\hspace{0.33em}\hspace{0.33em}\hspace{0.33em}\hspace{0.33em}\hspace{0.33em}\hspace{0.33em}\hspace{0.33em}\hspace{0.33em}\hspace{0.33em}\hspace{0.33em}\hspace{0.33em}\hspace{0.33em}\hspace{0.33em}\hspace{0.33em},\hspace{0.33em}\hspace{0.33em}\hspace{0.33em}\hspace{0.33em}\hspace{0.33em}\hspace{0.33em}\hspace{0.33em}\hspace{0.33em}\frac{\overline{\tau\:}}{\overline{\sigma\:}}>1\end{array}\right.$$

where $\:{\sigma\:}_{c}$ is the compressive strength, MPa; $\:\overline{\sigma\:}$ is the normal strength of the parallel connection, MPa; $\:\overline{\tau\:}$ is the tangential connection strength of the parallel connection, MPa; a=-0.965; b = 2.292; and c = 1.327.

Regression of tensile strength.

5 $$\:\frac{{\sigma\:}_{t}}{\overline{\sigma\:}}=\left\{\begin{array}{c}d{\left(\frac{\overline{\tau\:}}{\overline{\sigma\:}}\right)}^{2}+e\frac{\overline{\tau\:}}{\overline{\sigma\:}},\hspace{0.33em}\hspace{0.33em}\hspace{0.33em}\hspace{0.33em}0<\frac{\overline{\tau\:}}{\overline{\sigma\:}}\le\:1\\\:f\hspace{0.33em}\hspace{0.33em}\hspace{0.33em}\hspace{0.33em}\hspace{0.33em}\hspace{0.33em}\hspace{0.33em}\hspace{0.33em}\hspace{0.33em}\hspace{0.33em}\hspace{0.33em}\hspace{0.33em}\hspace{0.33em}\hspace{0.33em},\hspace{0.33em}\hspace{0.33em}\hspace{0.33em}\hspace{0.33em}\hspace{0.33em}\hspace{0.33em}\hspace{0.33em}\hspace{0.33em}\frac{\overline{\tau\:}}{\overline{\sigma\:}}>1\end{array}\right.$$

where $\:{\sigma\:}_{t}$ is the tensile strength, MPa; d=-0.174; e = 0.463; and f = 0.289.

Table 1 lists the values of the macroscopic and microscopic parameters of each stratum obtained based on the geological conditions of the working face.

**Table 1:** Macroscopic and microscopic physical and mechanical properties of strata.

Stratum	Lithology	Poisson’s ratio	Elastic modulus	Tensile strength /MPa	cohesion /MPa	Angle of internal friction /(°)
J15	Mudstone	0.15	7.2	1.36	6.32	39.4
J14	Fine-grained sandstone	0.21	24.8	3.06	5.77	38.8
J13	Gritstone	0.18	15.4	2.58	4.35	35.3
J12	Gritstone	0.18	15.4	2.58	4.35	35.3
J11	Siltstone	0.23	18.2	4.66	11.05	36.0
J10	Siltstone	0.23	18.2	4.66	11.05	36.0
J9	Coal	0.27	6.9	1.33	2.42	42.6
J8	Siltstone	0.23	18.2	4.66	11.05	36.0
J7	Coal	0.27	6.9	1.33	2.42	42.6
J6	Siltstone	0.23	18.2	4.66	11.05	36.0
J5	Coa	0.27	6.9	1.33	2.42	42.6
J4	Siltstone	0.23	18.2	4.66	11.05	36.0
J3	Coal	0.27	6.9	1.33	2.42	42.6
J2	Siltstone	0.23	18.2	4.66	11.05	36.0
J1	Medium-grained sandstone	0.18	15.4	2.58	4.35	35.3

#### Construction of PFC^3D^ model

To further analyze the delamination and fracture of the caved coal seam, the numerical model for the goaf of the WⅡ02040502 working face in Tun bao Coal Mine was constructed by the PFC^3D^ discrete element method. As shown in Fig. 1, the model is 500 m long, 249 m wide, and 677.15 m high. It is divided into 15 layers and the inclination of the coal seam is neglected. According to the coal seam mining height of 9.76 m. The particle size of the model ranges from 1 to 1.5 m. The measured particle size distribution of the core in the study area is referred to the particle size range of the core. The coal seam is excavated successively according to the working face operation procedure. Excavation is stopped when no more fractures are formed, and the next excavation is carried out. this process is repeated until the stop line is reached and a stable state is achieved. Table 1 lists the mechanical parameters of the rock strata.

#### Evolution characteristics of three zones and fractures in overburden of Goaf

Based on the numerical simulation scheme shown in Fig. 1 for coal seam mining, we studied the horizontal delamination fractures between the strata below the key stratum and the vertical damage fracture that penetrates the strata formed after the original rock stress is destroyed during coal seam excavation^11,12^. The collapse law of the overburden is explored and the collapse ranges in the three zones of overburden are accurately determined by tracking the dynamic collapses and fracture changes of the model.

**Fig. 2:**
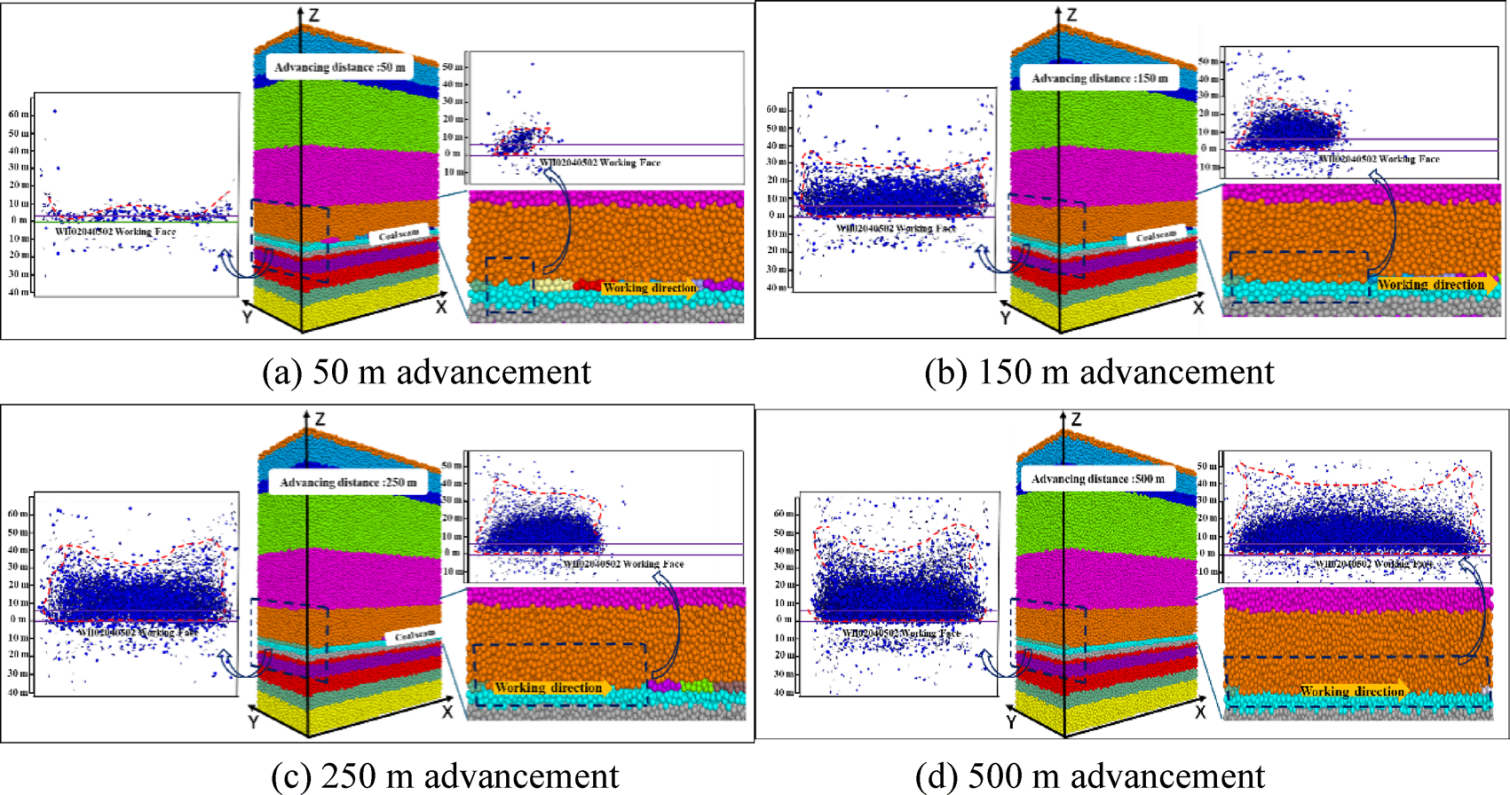
Simulation of goaf collapse and fracture development during coal seam caving.

The coal seam is divided into 10 sections along the mining direction, with each 50 m long. They are mined sequentially along the mining direction as shown in Fig. 2. The immediate roof remains and still functions as a support after 50 m advance. However, the roof is loosened, and a few fractures appear due to the disappearance of the immediate support force. After 150 m advance, the fractures develop to about 32 m high. Some fractures penetrate each other to form a network, and the fracture openings increase. Starting from the third section, the roof and overburden of the goaf in front collapse, the fractures further develop, and the number of fractures increase. Most of the fractures penetrate to form connected fractures and the fracture openings continuously increase, showing a trend of more on sides and less in the middle along the working face until the caving is completed.

**Fig. 3:**
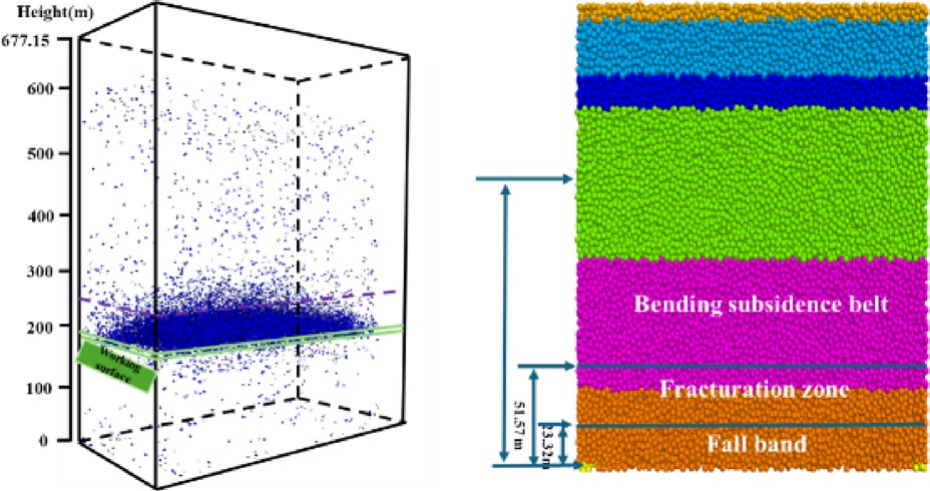
Schematic diagram for the damage and division of overburden.

The fractures develop to about 240 m long in the caving direction and 41 m high after 250 m advance and the number of fractures near the coal seam floor becomes larger as shown in Fig. 2(c). As the advancing distance reached 500 m, more fractures are formed on both sides along the working face and caving directions and fewer appear in the middle. The fracture distribution near the floor is also denser. The distribution of the fractures is segregated at the height of 23 m, with significantly more at the bottom and fewer in the upper (Fig. 3). Most of fractures in the bottom are connected and those in the upper are scattered and mostly closed. After the goaf collapse, the caving zone, fracture zone and bending subsidence zone are gradually formed in the overburden. Based on the division of ' three zones ' of overlying strata, the’Code for Hydrogeology, Engineering Geology and Environmental Geology Exploration of Coal Deposits ‘(GB/T12719-2022) and the commonly used quantitative criteria for discrete element simulation are strictly followed. According to the degree of fracture development and deformation, the thickness of the caving zone is about 23.32 m, and the thickness of the fracture zone is about 28.25 m. The above fracture development characteristics of overlying strata directly affect the pore distribution, and the next section will combine the porosity distribution map.

#### Variation law of porosity in Goaf

**Fig. 4:**
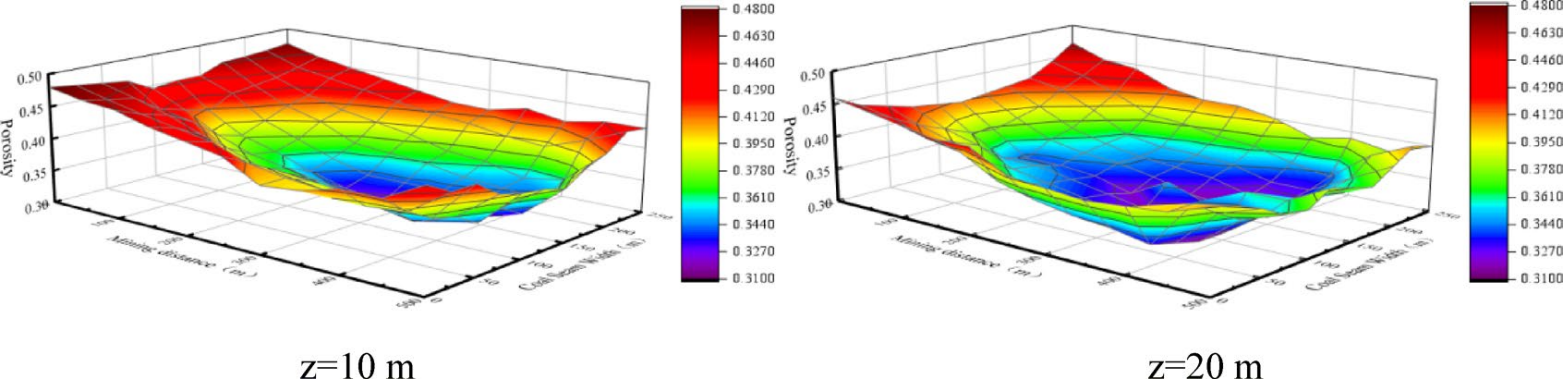
Surface diagrams of the porosity distributions in caving zone.

With the advancing of the working face, a large area of goaf composed of residual coal and collapsed rocks is left behind. The rocks in the caving zone are subject to the largest loading from the overburden, and the fracture deformation there is most significant. The irregular accumulation of collapsed rocks results in holes pores^13,14^ According to the visible crack transformation diagram in Fig. 4. In the caving zone, the cross sections at 10 m and 20 m from the working face floor, i.e. z = 10 and z = 20, are taken respectively, and the surface diagrams of their porosity distributions are shown in Fig. 4. As can be seen, the porosity distribution shows a “shovel” shape. The porosity decreases as it approaches the middle of the goaf.

**Fig. 5:**
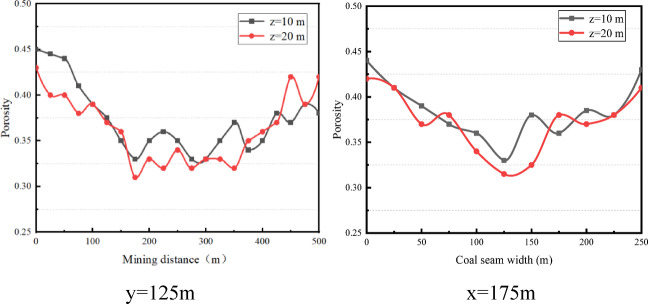
Line diagrams of the porosity distributions in caving zone.

The analysis of the porosity distributions along the caving and working face directions were analyzed suggests that the porosity is lowest in the middle of the goaf at 125 m in the working face direction and at 175 m in the mining direction. Therefore, the porosity distribution curves at the section of y = 125 m and x = 175 m in the stable state are selected for further analysis to better understand the fracture propagation characteristics and water seepage law in the overburden of goaf^15^. The collapsed coal rocks in the deep goaf tend to be compacted by the gravity of the overlying rocks, and the porosity reaches the minimum value at 175 m. As shown in Fig. 5, the porosity of the caving zone ranges from 0.31 to 0.33 in the stable and compacted area and is 0.43–0.45 in the naturally accumulated area.

**Fig. 6:**
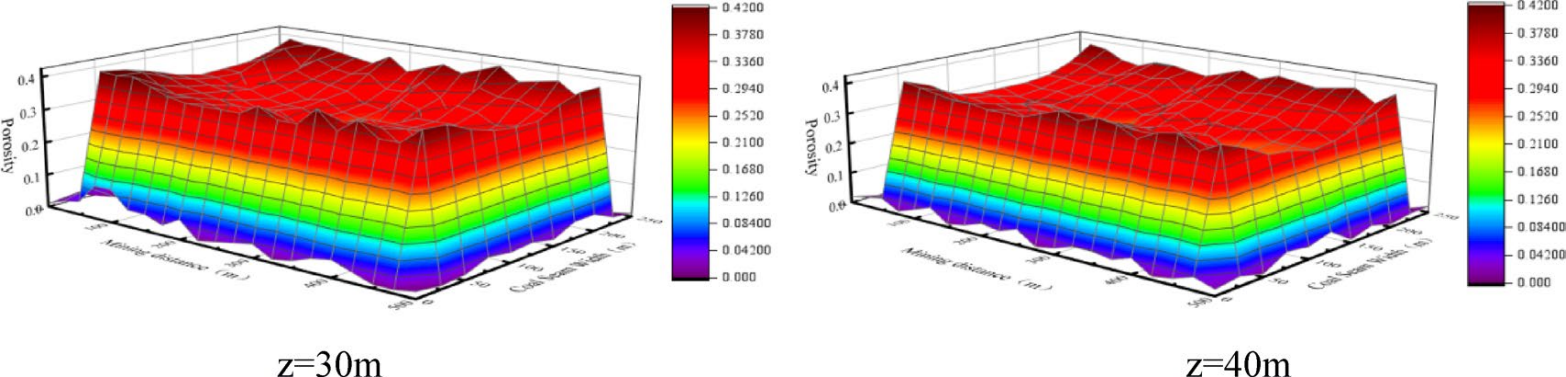
Surface diagrams of the porosity distributions in fracture zone.

**Fig. 7:**
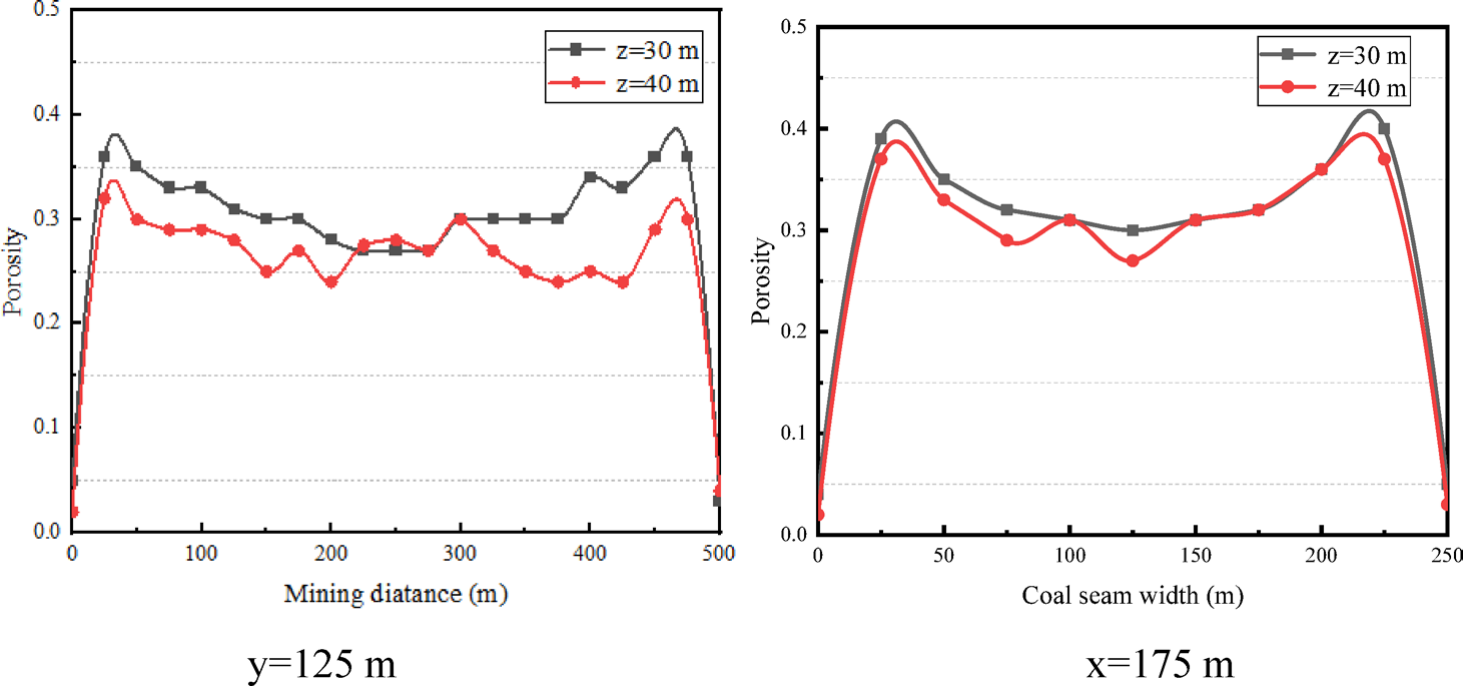
Line diagrams of porosity distributions in fracture zone.

The porosities on the cross sections at 30 m and 40 m from the working face floor, that is, z = 30 and z = 40, in the fracture zone are also analyzed^16–18^. As shown in Fig. 6 for the surface diagrams, the porosity increases first and then decreases along both the working face direction and the caving direction and shows the minimum value in the middle of the goaf. In addition, it can be seen from the line diagrams in Fig. 7 that the porosity of the fracture zone is 0.37–0.39 at the “peak” and 0.23 ~ 0.25 in the middle compacted area.

## Result verification and analysis

### Similar simulation experiment and verification

#### Experimental model construction

**Fig. 8:**
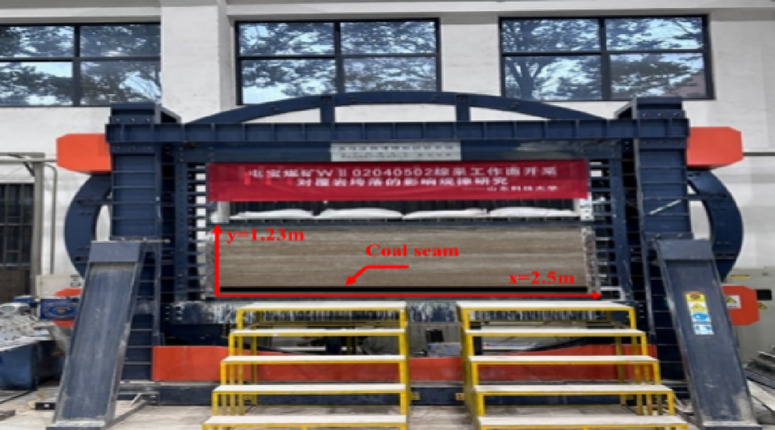
Photo of experiment device.

The physical similarity simulation experiment adopted a plane strain model frame with the size of 2.5 m × 0.4 m × 1.5 m (length × width × height). The geometric ($\:{\mathrm{{\alpha}}}_{\mathrm{L}}\mathrm{=}{\mathrm{L}}_{\mathrm{H}}/{\mathrm{L}}_{\mathrm{M}}$) and time (($\:{\mathrm{{\alpha}}}_{\mathrm{t}}\mathrm{=}\sqrt{{\mathrm{{\alpha}}}_{\mathrm{L}}}$)) similarity ratios were set to 1:240 and 1:15, respectively, based on the similarity theorem. The similarity ratios of the internal friction angle and Poisson’s ratio^19^ were 1. The stress similarity ratio ($\:{\mathrm{{\alpha}}}_{\mathrm{\sigma}}\mathrm{=}\frac{{\mathrm{r}}_{\mathrm{H}}}{{\mathrm{r}}_{\mathrm{M}}}{\mathrm{{\alpha}}}_{\mathrm{l}}$) and pressure similarity ratio ($\:{\mathrm{{\alpha}}}_{\mathrm{p}}\mathrm{=}\frac{{\mathrm{r}}_{\mathrm{H}}}{{\mathrm{r}}_{\mathrm{M}}}{{\mathrm{{\alpha}}}_{\mathrm{l}}}^{\mathrm{3}}$ ) were 1:300, and $\:\mathrm{1}/\mathrm{1.2}\times{\mathrm{10}}^{\mathrm{7}}$, respectively. The nterior dimension was 2.5 m×0.4 m×0.8 m (length×width×height). The lithology of the roof and floor is shown in Table 2, and some lithology experiments are shown in Table 3. Figure 8 shows the photo of the model frame. The compressive strength of the measured similar material is 1:300 of the prototype lithology, and the elastic modulus error is less than 5%, which meets the requirements of similar simulation.

**Table 2:** Roof and floor lithology and ratio.

Serial number	Top layer	Lithologic characters	Thickness of stratum/m	Elastic modulus	Poisson ratio	Tensile strength Pb-ten(MPa)	Angle of internal friction pb-fa(°)	Mass ratio River sand : gypsum : white powder : coal powder
1	main roof	siltstone	35	9.7	0.23	4.66	36	8:3:7:0
2	immediate roof	grit stone	24	15.90	0.15	3.13	42	7:5:5:0
3	coal	M4-5 coal	10	6.9	0.27	1.33	42.6	26:1:5:16
4	direct bottom	siltstone	12	35	9.7	0.23	4.66	8:3:7:0
5	Basic bottom	fine sandstone	12	24.8	0.21	3.06	38.8	7:4:6:0

#### Experimental data and result analysis

##### Identification and analysis of fracture evolution in overburden collapse

**Fig. 9:**
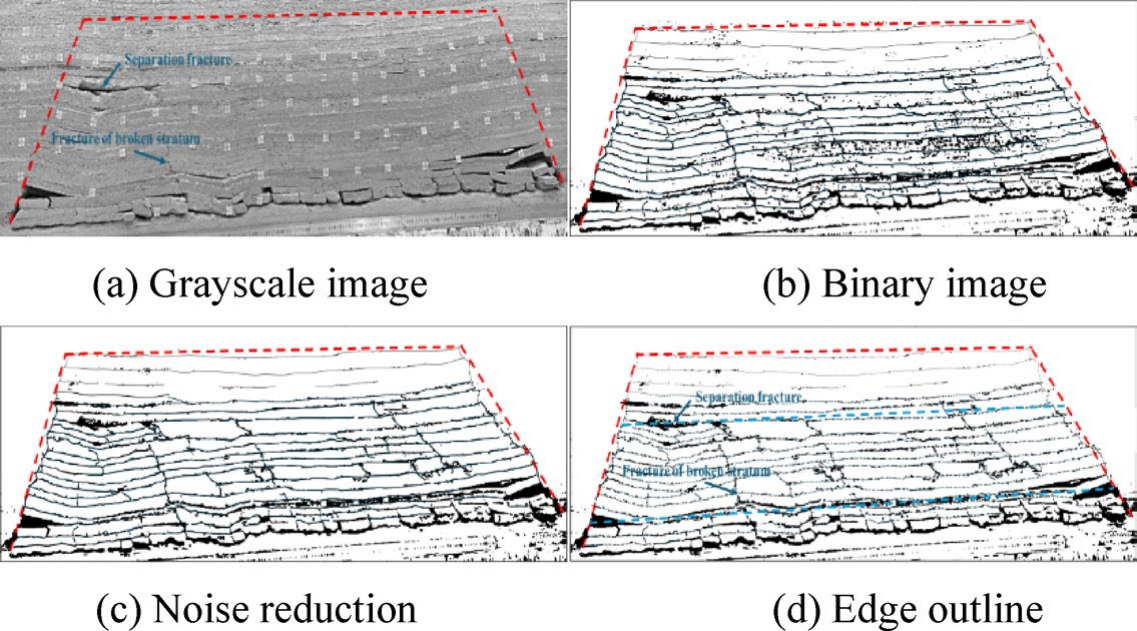
Identification and extraction of the fracture network in overburden.

**Fig. 10:**
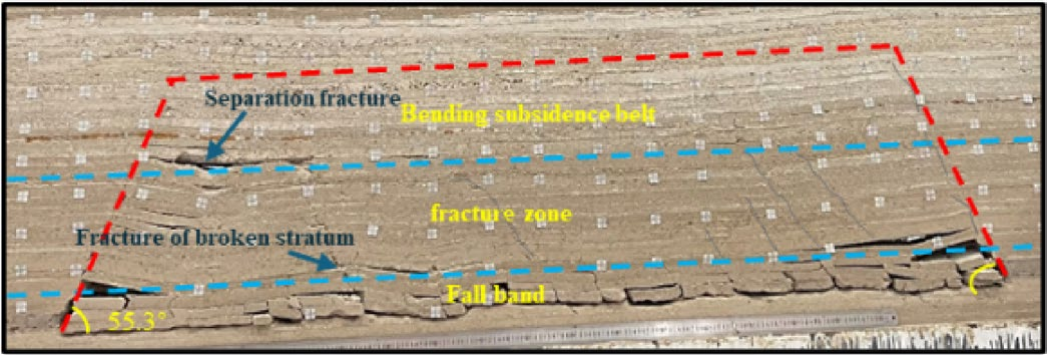
Distribution of fracture network in overburden.

The fractures in the obtained similar model image of overburden are identified and extracted using ImageJ software following the process detailed in Fig. 9. To facilitate the fracture identification and extraction, the image pixels are first converted into actual sizes for size marking^20^, and the image is converted into grayscale (black to white grayscale value range, 0-255). The binary black and white image is subjected to noise reduction processing to eliminate pseudo fractures, such as particle spots and fragments, using mean the filtering and opening-closing algorithm^21^, which results in a relatively clear fracture network outline. The extracted fracture network skeleton and edge outline can reflect the morphology and distribution characteristics of the fracture network in overburden^22^. The morphology and distribution characteristics of the fracture network in the overlying strata are shown in Fig. 10.

Fracture identification and extraction reveal a crisscrossed and interconnected fracture network that serves as a complex dominant pathway for water flow, providing critical insight into seepage mechanisms within overburden strata. A total of 132 fractures were identified, with a combined length of 20,736.15 mm and a total area of 87,519.95 mm², including 61 longitudinal fractures and 71 interlayer delamination fractures. The longitudinal fractures exhibit an average aperture of 4.21 mm and an average trace length of 67.54 mm, whereas the interlayer delamination fractures show averages of 5.40 mm in aperture and 75.53 mm in trace length.The longitudinal fractures, characterized by larger apertures and shorter traces, act as the primary seepage channels. In contrast, the interlayer delamination fractures, with longer traces and narrower openings, facilitate the connectivity and expansion of flow pathways. These statistical findings align with qualitative analyses of fracture distributions from original imagery, validating the reliability of the digital image^23^processing method applied. Integrating fracture network characteristics with analysis of dominant seepage channels offers a fundamental basis for understanding water seepage behavior in overburden systems^24^.

### Field observation and data analysis

#### Drilling arrangement and observation design

**Fig. 11:**
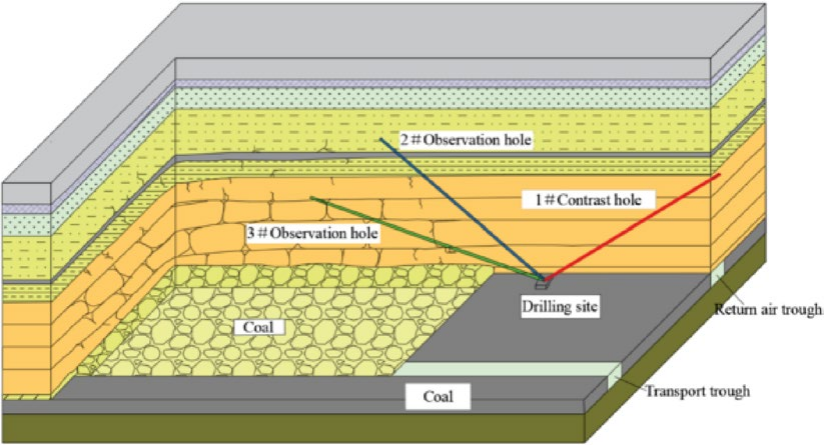
Schematic diagram of observation boreholes.

The field investigation of the “three zones” in goaf overburden was conducted on the WⅡ02040502 working face. The maximum height of the fracture zone after caving was calculated to be 49.7 m. Therefore, the vertical height of the water injection observation hole to the coal seam roof was designed^25,26^ to be 61 m. To avoid the influence of the original fractures on the test results, a reference borehole (1#) was created as the control to observe the water injection leakage flow of the rock stratum not affected by caving^27^. The 2 # and 3 # boreholes are test boreholes. The boreholes are arranged in the return air roadway of WII02040502 working face, 10 m away from the working face. Table 3 lists the parameters of these boreholes^28,29^, and their layout is shown in Fig. 11.

**Table 3:** Parameters of drilling holes.

Borehole	Upward angle/°	Azimuth	Diameter/mm	Depth /m	Height /m	Inclination/°
1#	25	N90	85	136	57.5	0
2#	25	N270	85	145	60.9	+ 10
3#	20	N270	85	167	56.8	-10

#### Field observation result analysis

**Fig. 12:**
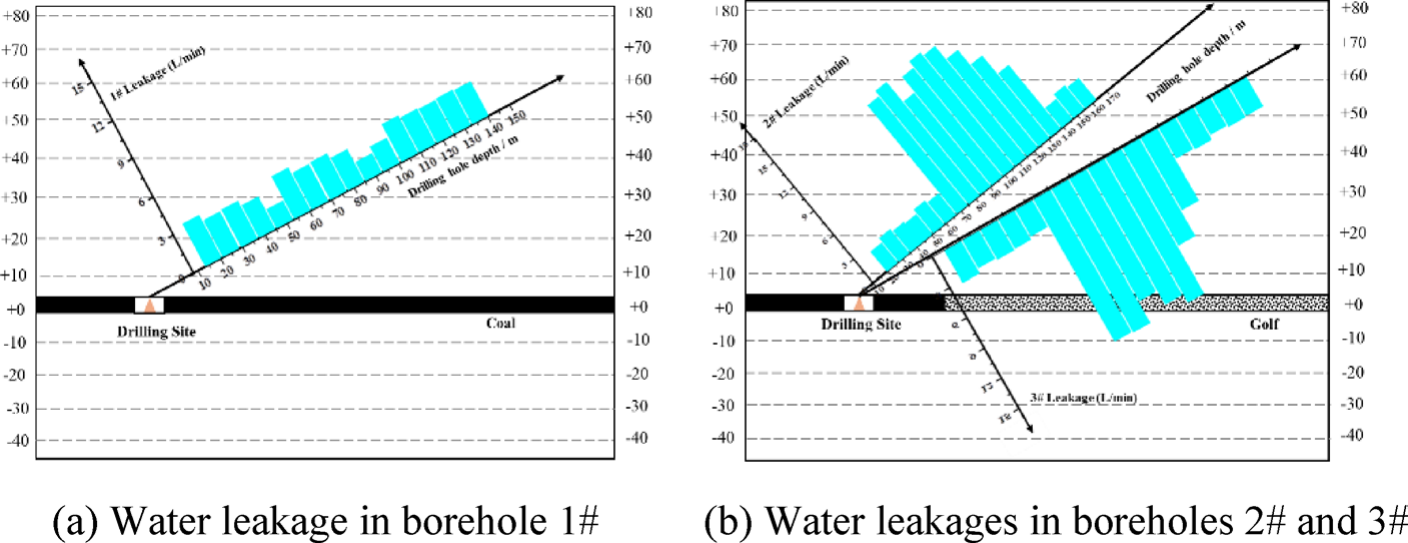
Schematic diagram for the relationship between water leakage and borehole depth.

Figure 12 shows water leakage measurements in inclined boreholes. In borehole #1 (vertical height: 23.9–57.1 m, Fig. 12a), located in the non-caved overburden, the average water loss was 2.72 L/min with minimal fluctuation, indicating uniformly developed primary fractures (Table 2).

In the # 2 borehole ( Fig. 12b ), at a depth of 72 m ( 26.3 m from the roof ), the leakage suddenly increased from 2.7 L / min to 14.8 L / min, and it was determined to enter the water-conducting fracture zone. At the depth of 133 m ( 47.8 m from the roof ), the leakage decreased from 11.2 L / min to 3.3 L / min, and the top boundary of the fracture zone was determined to be at 47.8 m. Borehole #3 exhibited a similar trend^30^: within 24.1–48.6 m, average leakage reached 12.32 L/min—significantly higher than in #1—reflecting intense fracturing within the fracture zone. From 48.6 to 56.8 m, average loss was 2.73 L/min, comparable to #1, indicating primarily primary fractures. Thus, the maximum height of the fracture zone is 47.5–48.6 m. The difference from PFC-simulated fracture height (51.57 m) is small, confirming simulation accuracy.

## Numerical simulation of water seepage in Goaf overburden fractures

### Model construction for water seepage

**Fig. 13:**
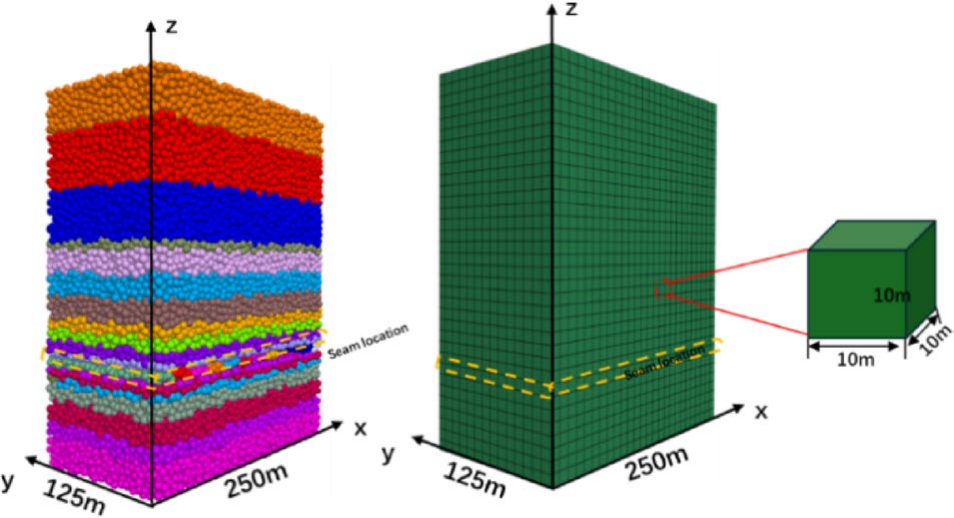
Model diagram.

Integrating the previously analyzed three-zone distribution characteristics of overlying strata with the geometric configuration parameters of fracture networks^31–33^, a coupled PFC-CFD computational framework was constructed as demonstrated in Fig. 13. The model configuration adopted linear contact mechanics with cubical grid elements measuring 10 m × 10 m × 10 m, culminating in a total discretization of 11,050 grid cells. The hydraulic boundary conditions were defined through water injection simulation at the uppermost stratigraphic cross-section corresponding to z = 340 m elevation, with interfacial contact forces calibrated to 50 MPa through iterative computational mechanics validation.

### Water seepage simulation result and law analysis

**Fig. 14:**
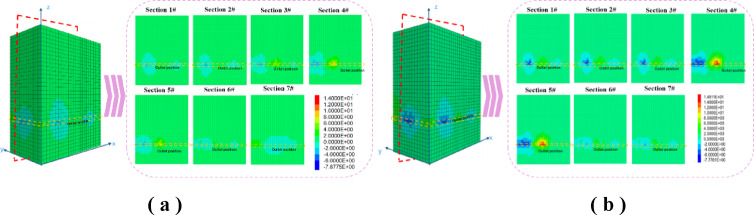
Water seepage pressure change distribution diagram. **(a)** Water injection rate 3.4 m^3^ / h pressure change cloud chart **(b)** Water injection rate 6.8 m^3^ / h pressure change cloud diagram

Figure 14 shows the evolution of water seepage pressure. Equidistant cross-sections parallel to the y-axis reveal a pronounced gradient between injection and outflow points, with nonlinear pressure attenuation. Increasing the injection rate from 3.4 m³/h to 6.8 m³/h raised the maximum pressure from 2.1 MPa to 4.3 MPa and accelerated gradient variation below z = 150 m, indicating stronger permeability anisotropy. A localized low-pressure zone at x = 125 m is likely due to model boundary effects and caved zone pore structure, consistent with known flow distortions from abrupt porosity^34–36^ changes.Seepage trajectories show fluid primarily follows pathways aligned with dominant fractures. After flow rate increase, these channels deviate by 15° from the NW–SE joint system, highlighting structural control on flow^37^. Secondary branches increased by 37.5%, forming reticulated networks within y = 60–90 m. This reorganization correlates with stress-enhanced pore connectivity, as supported by coupled stress-permeability mechanisms^38–40^.

The double increase of water injection rate leads to a super-linear relationship between Darcy flow rate and water injection rate. The average flow rate under the condition of 6.8 m^3^ / h is 2.3 times that of 3.4 m^3^ / h, which deviates from the predicted value of ideal seepage theory^41^, and reveals the inertial effect and local turbulent transition phenomenon caused by high flow rate. At the same time, the doubling of water injection rate and the erosion and reaming effect of high-speed water flow on micro-cracks increase the effective permeability by 18.7%. The pressure propagation range is expanded by 1.8 times, and obvious pressure disturbance is formed in the area of y >100 m, which indicates that there may be potential seepage instability risk in the edge coal pillar of goaf^42^.

**Fig. 15:**
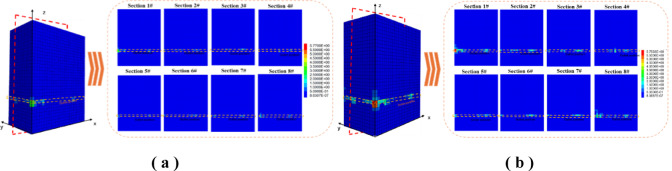
Change of water seepage velocity. **(a)** The change of water seepage velocity when the water injection rate is 3.4 m^3^ / h. **(b)** The change of water seepage velocity when the water injection rate is 6.8 m^3^ / h.

Figure 15 is a water seepage velocity change map, and eight water seepage velocity change profiles are intercepted equidistantly along the y-axis direction for analysis. The velocity cloud diagram along the y-axis equidistant section shows that the water seepage of the overlying strata in the goaf follows the composite mechanism of ' gradient drive-channel control-rate regulation ‘. The water flow is driven by water injection pressure and gravity. Before reaching the water outlet position, most of the area is in a low-speed dispersion state due to rock resistance. Only when the water flow migrates to the vicinity of the outlet position, the ' sink effect ' at the outlet end strengthens the local pressure difference and promotes the formation of high-speed concentrated flow in the dominant seepage zone.

The preferential flow zone around the mid-y-axis region acts as the hydrodynamic core controlling velocity distribution, where permeability heterogeneity dictates the spatial extent and magnitude of high-velocity regions. Fluid migrates preferentially along fracture-dominated pathways, forming distinct high-velocity channels. At an injection rate of 3.4 m³/h, the maximum velocity of 9.8 × 10⁻³ m/s occurs within the central region (y = 60–80 m), while peripheral areas exhibit velocities below 5 × 10⁻⁴ m/s due to limited permeability. Increasing the injection rate to 6.8 m³/h expands the main seepage zone from 15 m to 28 m in width and raises the maximum velocity to 1.6 × 10⁻² m/s, indicating a notable increase in velocity gradient. This behavior is closely associated with the heterogeneous porosity distribution of the fractured rock mass and the drag-dominated seepage mechanism.

Localized vortex structures observed in the overburden at x = 125 m and z = 90–140 m are likely caused by abrupt changes in pore connectivity between the caved and fractured zones. As shown in Fig. 15(b), velocity fluctuations within these vortices increase by 20% under higher injection rates, reflecting enhanced permeability anisotropy. These perturbations correspond to stress-induced porosity reconfiguration in fragmented rock masses, further supported by coupled hydromechanical simulations.

## Conclusions

This study investigates fracture development and water seepage in the overburden of the goaf at the WII02040502 working face of Tunbao Coal Mine through an integrated approach incorporating PFC simulation, similarity modeling, field monitoring, and ImageJ-based analysis. The main conclusions are as follows:

(1) The thicknesses of the caving zone and the fracture zone are determined to be 23.32 m and 28.25 m, respectively. Porosity in the caving zone ranges from 0.32 to 0.47, while within the fracture zone, it peaks at 0.38–0.40 on both sides of the goaf and decreases to 0.23–0.27 in the central region.

(2) A total of 132 fractures are identified, with a combined length of 20,736.15 mm and an area of 87,519.95 mm². These include 61 vertical fractures with average aperture and trace length of 4.21 mm and 67.54 mm, respectively, and 71 interlayer fractures averaging 5.40 mm in aperture and 75.53 mm in length. The vertical fractures serve as primary water seepage channels, whereas interlayer fractures facilitate connectivity and expansion of seepage pathways.

(3) The PFC-CFD coupled simulations reveal a compound seepage mechanism governed by pressure gradient, channel geometry, and flow rate. When the injection rate increases from 3.4 m³/h to 6.8 m³/h, the maximum pressure rises by 104.8% to 4.3 MPa. The main flow channel shifts approximately 12° toward the central axis and expands from 15 m to 28 m in width. Permeability anisotropy is significantly enhanced under high flow conditions due to particle erosion, with permeability coefficients along the joint direction being twice those in orthogonal directions.

research limitations:

(1) The PFC3 D model does not fully consider the factors such as natural heterogeneity of rock mass, random distribution of initial fractures and dip angle of coal seam. There is still room for improvement in the simulation accuracy of overburden collapse and fracture development, which is different from the complex geological environment on site. (2) Similar simulation is limited by similarity ratio and material properties, and the reduction degree of fracture evolution and seepage under complex stress is limited. The field observation relies on three borehole data, and the layout and quantity are insufficient. It is difficult to fully reflect the global characteristics of the spatial distribution and seepage parameters of the ' three zones ‘, and there may be local data deviations.

Suggestions for future research:

(1)Incorporating key geological factors such as rock mass heterogeneity, initial fracture distribution, and coal seam dip angle, a more refined particle contact model is introduced to improve the simulation authenticity of overburden rock collapse and fracture evolution. Combined with CT scanning and other technologies, a three-dimensional real pore-fracture model is constructed to enhance the accuracy of seepage simulation.(2) The high similarity ratio and high performance similar materials are developed, the experimental loading method is improved, and the overburden failure and seepage process under complex stress paths are simulated. Increase the number of on-site observation boreholes, optimize the layout of boreholes, and combine distributed optical fiber sensing technology to obtain global and dynamic seepage and fracture development data. 

## Data Availability

The datasets generated and analyzed during the current study are available from the corresponding author on reasonable request.
